# Using a Gene Network of Pyroptosis to Quantify the Responses to Immunotherapy and Prognosis for Neuroblastoma Patients

**DOI:** 10.3389/fimmu.2022.845757

**Published:** 2022-03-24

**Authors:** Bingying Luo, Limin Wang, Weijing Gao, Yudong Su, Yao Lu, Jian Zheng, Jie Yin, Qiang Zhao, Jie Li, Yurong Da, Long Li

**Affiliations:** ^1^Key Laboratory of Immune Microenvironment and Diseases of Educational Ministry of China, The Province and Ministry Co-sponsored Collaborative Innovation Center for Medical Epigenetics, Department of Immunology, Tianjin Medical University, Tianjin, China; ^2^Key Laboratory of Cancer Prevention and Therapy, Tianjin Clinical Research Center for Cancer, Department of Pediatric Oncology, Tianjin Medical University Cancer Institute and Hospital, National Clinical Research Center for Cancer, Tianjin, China

**Keywords:** neuroblastoma, pyroptosis, immunotherapy, prognosis, etoposide

## Abstract

**Background:**

Pyroptosis, as an inflammatory form of cell death, is involved in many physiological and pathological processes. Neuroblastoma is the most common extra-cranial solid tumor in children. In this study, the relationship between pyroptosis and tumor microenvironment in neuroblastoma was systematically studied.

**Methods:**

We integrated four datasets of neuroblastomas. Through robust clustering of the mRNA expression profiles of 24 pyroptosis-related genes, a total of three pyroptosis patterns were identified. We then constructed a novel scoring method named as pyroscore to quantify the level of pyroptosis in neuroblastoma. Multi-omics data and single-cell RNA sequencing were used to accurately and comprehensively evaluate the effectiveness of pyroscore. Clinical data sets were used to evaluate the use of pyroscore to predict the responsiveness of immune checkpoint treatment.

**Results:**

High pyroscore was associated with good prognosis, immune activation, and increased response to checkpoint blockade immunotherapy. Multivariate Cox analysis revealed that the pyroscore was an independent prognostic biomarker and could increase the accuracy of clinical prediction models. Etoposide, a drug picked up by our analysis, could increase the sensitivity of neuroblastoma cells to pyroptosis. External verification using four cohorts of patients who had received immunotherapy showed that high pyroscore was significantly associated with immunotherapy treatment benefit.

**Conclusions:**

Taken together, this study revealed that pyroptosis-related gene network could quantify the response of neuroblastoma to immune checkpoint blockade therapy and prognosis, and it may be helpful for clinical practitioners to choose treatment strategies for neuroblastoma patients.

## Introduction

Neuroblastoma (NB) is an embryonic tumor that arises from the developing sympathetic nervous system. It is the most common extra-cranial solid tumor of childhood and the most common cancer in the first year of life, occupying up to 13% of all pediatric cancer fatalities (1, 2). During the past 30 years, increasingly revolutionary, intensive therapeutic strategies have been developed to treat NB patients (3), and immunotherapy is a particularly promising therapy for fighting against NB (4–7). Although immunotherapy has shown exciting therapeutic prospect, not all patients receiving immunotherapy benefit from it (8, 9). The complexity and heterogeneity of tumor microenvironment (TME) is one of the factors that influence the therapeutic effect. The TME in NB includes not only tumor cells but also vascular endothelial cells, cancer-associated fibroblasts (CAFs), mesenchymal stromal cells (MSCs), Schwann cells, and infiltrating immune cells (10). Extensive studies have revealed that the TME could influence the progression of NB and the response rate to immunotherapy (11). Tumor-infiltrating immune cells can even indicate the prognosis of NB (12, 13).

Pyroptosis is defined as gasdermin-mediated programmed necrosis (14, 15). A great deal of evidence shows that pyroptosis can affect the development of tumors (16, 17). More and more studies have proved that pyroptosis plays an essential role in tumor cell proliferation, invasion, and metastasis, hence affecting the prognosis of cancer. However, the relationship between pyroptosis and tumors is diverse in different tissues and genetic backgrounds (18).

To date, the role of pyroptosis-related genes in neuroblastoma remains unclear. Herein, we identified three patterns of pyroptosis among 964 tumors from patients with neuroblastoma and systematically associated them with pathological features and immune cell infiltration. We then developed pyroptosis score (pyroscore) to quantify pyroptosis patterns. Finally, four immunotherapy cohorts were used to confirm that patients with higher pyroscore was associated with significant therapeutic advantages and clinical benefits. Thus, the pyroscore is proved to be a powerful prognostic biomarker and an accurate predictor for responsiveness to immunotherapy, and it may be helpful to guide clinical medication for neuroblastoma patients.

## Materials and Methods

### Neuroblastoma Data Sets and Data Preprocessing

We collected four neuroblastoma expression profiles containing survival data from public databases: TARGET-NB (RNA-seq), GSE49710 (microarray), GSE16476 (microarray), and E-MTAB-8248 (microarray). Altogether, 964 NB samples with normalized gene expression and clinical information were procured for further analysis. The Therapeutically Applicable Research to Generate Effective Treatments (TARGET) data are hosted by Genomic Data Commons (GDC); therefore, the neuroblastoma data TARGET-NB was downloaded from the GDC Data Portal (https://portal.gdc.cancer.gov/). The raw data of GSE49710 and GSE16476 were downloaded from the Gene Expression Omnibus (GEO; https://www.ncbi.nlm.nih.gov/geo/). The raw data of E-MTAB-8248 was downloaded from ArrayExpress database (https://www.ebi.ac.uk/arrayexpress/). In order to make the TARGET-NB data more consistent with the other three microarray data sets, we converted fragments per kilobase of transcript per million fragments mapped (FPKM) to transcripts per million (TPM) values (19). The combat algorithm of the sva R package (20) was used to integrate all samples. Single-cell RNA-seq data of neuroblastoma was obtained from GSE137802. The workflow of this article is shown in a diagram (**Supplementary Figure S1**). It provides an overview of procedures used in this project.

### Consensus Clustering for Pyroptosis-Related Genes

A consensus clustering algorithm (based on Euclidean distance and Ward’s linkage) was applied to determine the number of clusters of 24 pyroptosis-related genes (21) in meta cohort, and the optimal cluster number was determined through the proportion of ambiguous clustering (PAC) algorithm. The clustering process was completed by the ConsensuClusterPlus R package (22) and iterated 1,000 times to ensure the stability of the results.

### Immune Infiltration Inference

We used three algorithms to infer tumor immune infiltration: ESTIMATE (23), CIBERSORT (24), and ssGSEA (25). CIBERSORT is a tool for deconvolution of the expression matrix of human leukocyte subtypes from bulk tissue gene expression profiles based on the principle of linear support vector regression. A 547 signature genes matrix containing 22 functionally defined human immune subsets (LM22) profiles was provided as input. The data were uploaded to the CIBERSORT web portal (http://cibersort.stanford.edu/) and iterated 1,000 times to get the results. ESTIMATE is a tool that uses gene expression signatures to infer the fraction of stromal and immune cells in tumor samples. The score derived from ssGSEA reflects the degree to which the input immune gene signature is coordinately up- or downregulated within a sample.

### Dimension Reduction and Generation of Pyroscore

In order to obtain the differentially expressed genes (DEGs) of different pyroptosis groups, we carried out the following steps: first, we conducted a pairwise difference analysis among the three groups using limma package (26) and took the intersection to get the preliminary gene list. DEGs were determined by the limma package, which used linear modeling and empirical Bayesian methods to obtain posterior variance estimates. |log_2_FC|>1 and adjusted p<0.05 (Benjamini–Hochberg correction) were used to set the threshold. Then, we divided these genes into positively or negatively related gene sets. Finally, the Boruta algorithm was used to perform dimension reduction to obtain the de-redundant DEGs.

For the DEGs expression profile, the first principal component was extracted to serve as the signature score. Principal component analysis (PCA) transforms the original data into a new coordinate system through orthogonal linear transformation. The first principal component explains the maximum variance of the original data and removes noise and redundancy while retaining important information. The final pyroscore of each sample was calculated by the following formula:


$$\text{Pyroscore}=\Sigma {\text{PC}1}_{\text{GPPGs}}-\Sigma {\text{PC}1}_{\text{BPPGs}}$$

### Functional and Pathway Enrichment Analysis

Over-representation analysis (ORA) was used to determine the enrichment pathway by using a list of genes through the clusterProfiler R package (27). Gene ontology (GO) terms with adjusted p-values <0.05 were selected. In addition, gene set enrichment analysis (GSEA) (28) was used to detect global changes of all genes detected, so it can identify small but coordinated ways. The enrichplot R package (https://github.com/GuangchuangYu/enrichplot) was used to visualize the results. At the same time, gene set variation analysis (GSVA) was used to calculate the score of the biological process gene set constructed by Mariathasan et al. (29).

### Genomic Data Collection and Somatic Mutation

The TARGET-NB project included genome sequencing data. These files were downloaded through GDC Data Portal (https://portal.gdc.cancer.gov/). The somatic mutation landscape and the mutation frequency of each gene of the high and low pyroptosis group was realized by the maftool R package (30).

### Drug Sensitivity Prediction

In order to predict the possible effective chemotherapy drugs, we collected the drug sensitivity data from two databases: Cancer Therapeutics Response Portal (CTRP v.2.0) and PRISM Repurposing dataset (19Q4). CTRP and PRISM provide the area under the dose–response curve (area under the curve, AUC) values as a measure of drug sensitivity (31). We used the pRRophetic R package (32) to construct a ridge regression model to predict the AUC value according to cell line gene expression profile of two databases and the meta cohort gene expression profile. The role of candidate drugs was further evaluated by the Connectivity Map (CMap) and displayed therapeutic targets. By comparing the DEGs with the reference data sets, the correlation score (−100–100) was obtained. Negative values indicated the potential therapeutic effects of the drug.

### Immune Checkpoint Treatment Response

Tumor Immune Dysfunction and Exclusion (TIDE) (13) is an algorithm for predicting immunotherapy response. It is a computational framework developed to evaluate the potential of tumor immune escape from the gene expression profiles of cancer samples. The expression profile was uploaded to the website of TIDE (http://tide.dfci.harvard.edu/), and the immunotherapy response (response or not response) was predicted. The website’s results also yielded immune dysfunction score and immune exclusion score, MDSC score, etc. to evaluate the robustness of immunotherapy response and immune evasion mechanisms comprehensively. Submap inferred drug responsiveness by comparing transcriptome similarities between the NB cohort and a cohort receiving immunotherapy.

### Cell Culture and Pyroptosis Assays

Neuroblastoma cell line SH-SY5Y was cultured and treated with dimethyl sulfoxide (DMSO) (control) or 30 μm/60 μm etoposide (48 h) in Dulbecco’s modified Eagle’s medium (DMEM) supplemented with 10% fetal bovine serum, penicillin–streptomycin–glutamine, nonessential amino acids, sodium pyruvate, and 2-mercaptoethanol to induce cell death. The pyroptosis state of the cells was determined by microscopy and lactate dehydrogenase (LDH) release assay using the LDH Cytotoxicity Assay Kit (C0017; Beyotime) according to the manufacturer’s protocol. The percentage of LDH release was calculated as follows: % LDH release = (compound-treated LDH activity − spontaneous LDH activity) (maximum LDH activity − spontaneous LDH activity) − 1 × 100. Calcein-AM staining was used to quantify living and dead cells for the cell death assay. Calcein-AM can easily penetrate the cell membrane and then form calcein, which remains in the cell and observed as green fluorescence. Pyroptotic cells form membrane pores; then, the green fluorescence disappears. SH-SY5Y cells were stained with 2 μM calcein-AM. The images were acquired using a fluorescence microscope. Western blot analysis was performed using antibodies against Caspase-3 (CST, 9662S), DFNA5/GSDME (Abcam, ab215191), and GAPDH (Beyotime, AF1186).

### Transcriptome and Clinical Data Sets With Immune Checkpoint Blockade

Four cohorts (IMvigor210, GSE91061, GSE78220, and GSE35640) that had received immune checkpoint blockade (ICB) treatment were downloaded to verify the predictive value of the pyroscore. In IMvigor210, patients with metastatic urothelial cancer were treated with an anti-PD-L1 agent (Atezolizumab). The expression profile and clinical data are packaged into R package CoreBiologies (29) based on the Creative Commons 3.0 License. GSE91061, GSE78220, and GSE35640 were download from GEO. In GSE91061, patients with advanced melanoma were treated with anti-PD-1 agent (Nivolumab). In GSE78220, patients with metastatic melanoma were treated with anti-PD-1 agent. In GSE35640, patients with metastatic melanoma were treated with MAGE A3 immunotherapeutic.

### Statistical Analysis

All statistical methods were executed on R software (v4.0.2). For the comparison of two or more continuous variables, the unpaired Student’s t-test was performed for the data that obey the normal distribution, and the Wilcoxon test or Kruskal–Wallis test was performed for non-normally distributed data. Correlation of two variables was measured by Spearman’s rank-order correlation. Two-sided Fisher’s exact test was used to measure whether there was a difference in the rate between groups. We standardized the data by the z-score method before their compilation. Gene ordering for GSVA is based on the log_2_FC between two pyroptosis groups. Survival analysis was performed by the survival package. Log-rank test was used to determine whether there was a difference in survival time between groups. The appropriate cut point was determined by the X-tile software. The univariate and multivariate Cox regression model was used to determine independent prognostic factors by using the survminer package, and the results was visualized by ggforest package. Two-tailed *p*<0.05 was considered as statistically significant.

## Results

### Determination of the Association Between Pyroptosis-Related Genes and Prognosis in NB

**Figure 1:**
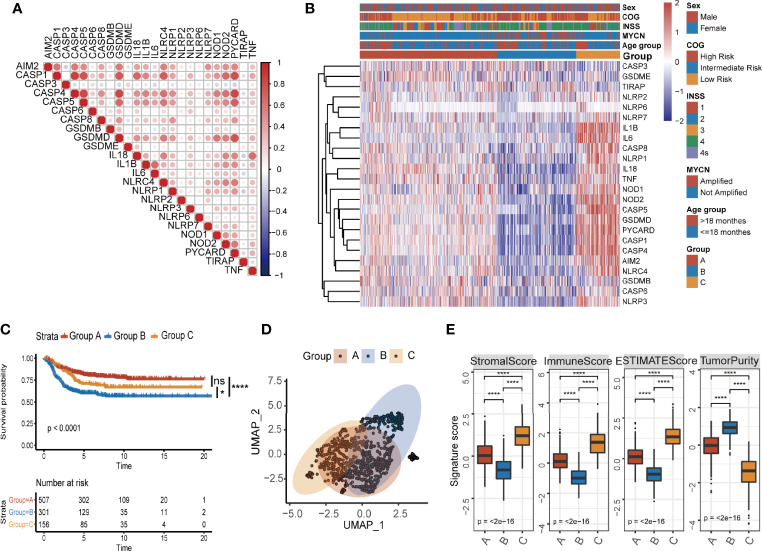
Identification of three pyroptosis groups with different survival status and immune infiltration conditions in NB. **(A)** Correlation of mRNA expression of 27 pyroptosis-related genes. **(B)** Consensus clustering heatmap of 27 pyroptosis-related genes. **(C)** Kaplan–Meier curves for overall survival (OS) of all NB patients with pyroptosis groups (log-rank test, *p* < 0.0001, * *p* < 0.05; **** *p* < 0.0001; ns, not significant). **(D)** UMAP of the mRNA expression profiles of pyroptosis-related genes from the NB patients in the meta cohort. **(E)** The value of ESTIMATE score in three pyroptosis groups (Kruskal–Wallis test, ****p < 0.0001). The thick line represents median value. The bottom and top of the boxes are the 25th and 75th percentiles (interquartile range).

In order to evaluate the possibility that pyroptosis-related genes could be used to predict prognosis of NB patients, we identified a total of 24 pyroptosis-related genes (21). These genes include NLRs and inflammasome adaptors (NLRP3, PYCARD, etc.), caspases (CASP3, CASP8, etc.), gasdermins (GSDMB, GSDMD, and GSDME), and pro-inflammatory cytokines (IL1B, IL6, TNF, etc.), which is a comprehensive list that reflects levels of pyroptosis. We then collected data of 964 NB patients with survival time from four data sets (TARGET-NB, GSE49710, GSE16476, and E-MTAB-8248) and formed a meta-cohort. When the 24 pyroptosis-related genes were run against each other in the data sets, we found a good positive correlation between these genes (**Figure 1A**), which indicates that these genes may play the same role together. Next, we performed unsupervised consensus clustering for mRNA expression profiles of the 24 pyroptosis-related genes in the meta-cohort and generated three groups of patients (**Figure 1B**; **Supplementary Figures S2A, SB**) with group B having the lowest overall expression of the pyroptosis-related genes. The three groups identified above had significant differences in survival (**Figure 1C**) with group B having the lowest survival probability. To further explore the molecular basis and visualize the relationship among the three groups defined by the pyroptosis-related genes, samples were reduced to two dimensions using Uniform Manifold Approximation and Projection (UMAP) analysis based on the top 3,000 most variable protein-coding genes. The results confirmed that the three groups defined by the pyroptosis-related genes can be clearly distinguished (**Figure 1D**).

Next, we explored the tumor microenvironment in the three groups. We used the ESTIMATE algorithm to infer immune scores and tumor purity for the three groups (**Supplementary Table S1**). Pyroptosis group B had the lowest immune score and the highest tumor purity (**Figure 1E**). To assess immune cell infiltration in detail, we used two algorithms: CIBERSORT and ssGSEA. The results of CIBERSORT are organized into four categories (33): total lymphocytes, total dendritic cell, total macrophage, and total mast cell. Pyroptosis group B, which had the lowest overall expression of pyroptosis-related genes and the lowest survival probability, was characterized by a significantly lower density of lymphocytes and a significantly higher infiltration of macrophages and total mast cells (**Supplementary Figure S2C**). The results of both CIBERSORT and ssGSEA further showed that pyroptosis group B exhibited low infiltration of CD8^+^ T cells, memory resting CD4^+^ T cells, and activated NK cells, while the scores for inhibitory cells such as Tregs were higher (**Supplementary Figures S2D, E**). The overall inactive/inhibitory immune microenvironment of tumors in group B was consistent with the lowest survival probability of patients in the group. The results clearly showed that there is an association with pyroptosis and active state of immune microenvironment in NB patients.

### Development of the Pyroptosis Signature and Functional Annotation

**Figure 2:**
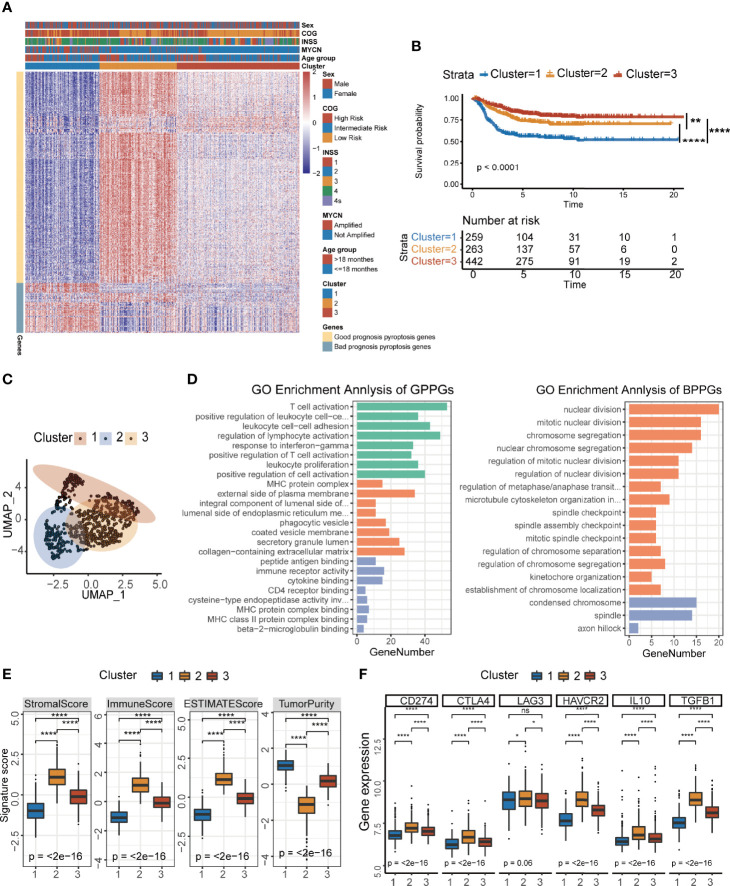
Development of the pyroptosis signature and functional annotation. **(A)** Consensus clustering of common DEGs among three pyroptosis groups to classify patients into three clusters: Clusters 1–3. **(B)** Kaplan–Meier curves of OS time for the three clusters of patients (log-rank test, *p* < 0.0001, ** *p* < 0.01; **** *p* < 0.0001). **(C)** UMAP plot of three clusters of patients. **(D)** GO enrichment analysis of the two pyroptosis-relevant signature genes: GPPGs on the left, BPPGs on the right. The x-axis indicates the number of genes within each GO term. **(E)** The value of ESTIMATE score in three clusters of patients (Kruskal–Wallis test, ****p < 0.0001). **(F)** Immune-checkpoint-relevant gene expression in three clusters of patients (Kruskal–Wallis test. *p < 0.05; ****p < 0.0001; ns, not significant).

To identify the underlying biological characteristics of each pyroptosis group, we extracted the DEGs among the three groups through the limma package. A total of 910 DEGs had been identified, which were used to cluster patients at genetic level (**Supplementary Figures S3A–C**, **Supplementary Table S2**). Venn diagram showed the number of DEGs among the three clusters (**Supplementary Figure S3D**). We visualized the changes between pyroptosis groups identified in the previous section (**Figure 1B**) and the newly generated clusters using a Sankey diagram (**Supplementary Figure S4A**) and found that favorable consistency between the two sets of grouping could be confirmed (χ^2^ tests, *p*<2.2×10^−16^). Next, we applied the Boruta algorithm to reduce the dimensionality and de-redundancy of DEGs. A total of 436 most representative genes out of 910 DEGs were identified finally and then used to construct our scoring system. We divided the 436 most representative genes into two signatures according to their correlations with the clusters (**Figure 2A**). The genes that were positively related to the clusters were named as good prognosis pyroptosis genes (GPPGs, 352 genes) because their expressions were higher in clusters 2 and 3 that had much better overall survival compared with cluster 1 (**Figure 2B**), while the genes that enriched in cluster 1 that had the lowest overall survival were termed as bad prognosis pyroptosis genes (BPPGs, 84 genes) (**Supplementary Table S3**). Hierarchical clustering results confirmed the division between GPPGs and BPPGs (**Supplementary Figure S4B**). The results of survival analysis shown by the Kaplan–Meier plotter suggested that there were considerable differences among the three clusters of patients. The patients in cluster 1 had the shortest overall survival compared to those of the other two clusters (log-rank test, cluster 1 vs. cluster 2, *p*<0.0001; cluster 1 vs. cluster 3, *p*<0.0001; **Figure 2B**). UMAP analysis confirmed that the three clusters were separated (**Figure 2C**).

In order to systematically explain the biological functions of GPPGs and BPPGs, GO analysis was performed by the clusterProfiler R package (**Supplementary Table S4**). The GPPGs, which were positively correlated with clusters 2 and 3 that had better prognosis were mainly enriched in the immune-related pathways, especially for the activation of T cells and antigen processing and presentation (**Figure 2D**). On the contrary, BPPGs were enriched in the regulation of cell cycles, which are essential for tumor progression and deterioration (**Figure 2D**). We checked the relationship between pyroptosis-related genes (24 genes in **Figure 1A**) and the final 436 DEGs that defined GPPGs and BPPGs and found seven genes in common. There was an overlap between the pyroptosis-related genes and the DEGs enriched in immune-related pathways (**Supplementary Figure S4C**). Expression pattern analysis of the three clusters revealed that CASP1, CASP4, CASP5, GSDMD, NLRP3, and PYCARD had the same expression pattern as GPPGs, and IL6 had the same expression pattern as BPPGs (**Supplementary Figure S4D**). ESTIMATE results showed that cluster 1 had the lowest immune score and the highest tumor purity (**Supplementary Figure 2E**). Correspondingly, samples in the three clusters exhibited different immune cell infiltration landscapes (**Supplementary Figures S4E, F**). Cluster 1 had the lowest infiltration of CD8^+^ T cells, memory resting CD4^+^ T cells, and activated NK cells, and had an increase in macrophages, which resembles the “immune desert” phenotype reported previously (34). Cluster 2 was characterized as the “immune exhausted” phenotype (35) because it exhibited the highest lymphocyte infiltration; however, the expressions of molecules that are essential for T-cell exhaustion, including PDCD1 (PD-1), CD274 (PD-L1), CTLA4, and LAG3, were higher as well (**Figure 2F**).

### Construction of Pyroscore Based on PCA

**Figure 3:**
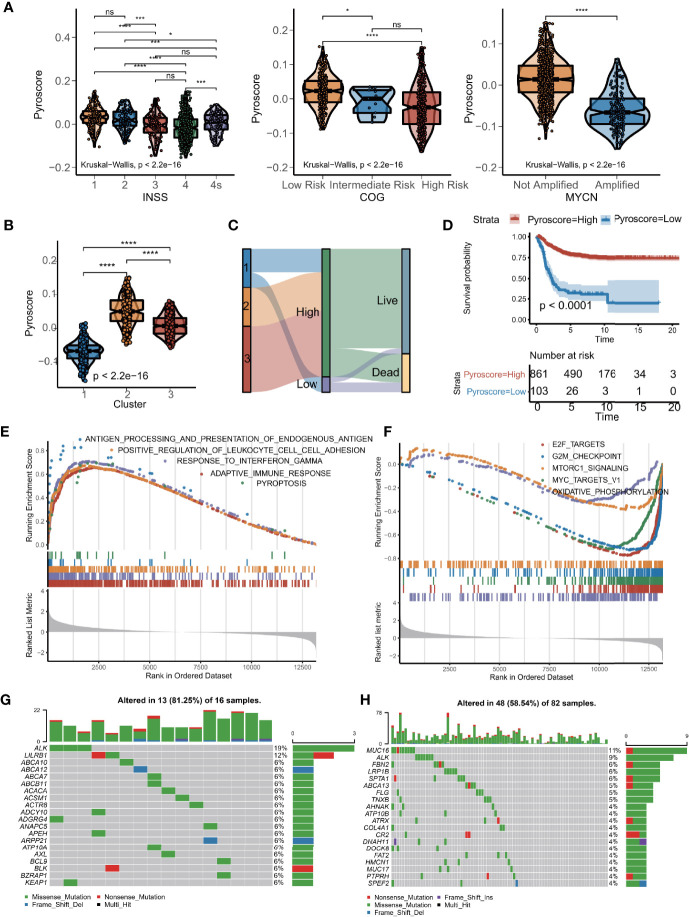
Construction of pyroscore based on PCA. **(A)** Violin diagram of pyroscore levels of different clinical features (Kruskal–Wallis test, *p < 0.05; **p < 0.01; ****p < 0.0001; ns, not significant). **(B)** Violin diagram of pyroscore levels among the three clusters of patients (Kruskal–Wallis test, ****p < 0.0001). **(C)** Sankey diagram of pyroptosis clusters in groups with pyroscore and survival outcomes. **(D)** Kaplan–Meier curves for high and low pyroscore patient groups (log-rank test, *p* < 0.0001). **(E)** GSEA identified immunity-related pathways enriched in the high pyroscore group. **(F)** GSEA showed related pathways enriched in the low pyroscore group. **(G, H)** Comparisons of the mutation landscape in the TARGET-NB cohort between groups with low and high pyroscore.

To develop a precise and brief system to measure the genetic interaction network that could explain the correlation of pyroptosis to prognosis in NB, the expression data of the 436 DEGs that defined GPPGs and BPPGs were used to construct a scoring system. We implemented the PCA algorithm to get an individual score named as pyroscore. According to the two gene signatures (GPPGs and BPPGs) mentioned above, the first principal components were taken, respectively. We first tested pathway signatures in the NB dataset to characterize the roles of ΣPC1_GPPGs_ and ΣPC1_BPPGs_ individually. It can be observed that ΣPC1_GPPGs_ and ΣPC1_BPPGs_ were related to different pathways, such as ΣPC1_GPPGs_ was mainly related to immunity, while ΣPC1_BPPGs_ was mainly related to pathways that are beneficial to tumor development including glycolysis and Wnt (**Supplementary Figure S5A**). The pyroscore was calculated by subtracting ΣPC1_BPPGs_ from ΣPC1_GPPGs_, and pyroscore score was much better than ΣPC1GPPGs and ΣPC1BPPGs alone to predict prognosis (**Supplementary Figures S5B–D**). The final value of pyroscore ranged from −0.155 to 0.152. After computing the quantitative score of each patient, we observed significant differences in the pyroscore among the three clusters of patients. International Neuroblastoma Staging System (INSS), Children’s Oncology Group (COG), and *MYCN* amplification have been used clinically to determine the treatment plan and predict prognosis for NB patients. Grouping by clinical characteristics (INSS, COG, and *MYCN* amplification), the results revealed that higher pyroscore was associated with milder disease state (**Figure 3A**). For example, the pyroscore levels of tumors in INSS 1–4 gradually decrease, and the pyroscore levels of patients in INSS 4S, which was generally considered to have a better prognosis, were higher than those of the INSS 4 stage. The pyroscore inversely correlated with COG staging as well. Cluster 1, which has the worst prognosis, had the lowest pyroscore (**Figure 3B**).

We then used the X-tile software to divide the meta-cohort based on the pyroscore of each sample. Sankey diagram was used to visualize the association of patients with pyroscore and the corresponding prognosis (**Figure 3C**). Patients who scored high had significant different clinical characteristics from those with low pyroscore (**Supplementary Figure S6A**), especially in terms of frequency of *MYCN* amplifications, which is one of the most important molecular drivers for NB. Furthermore, the patients with high pyroscore levels also had higher survival probability (log-rank test, *p*<0.0001; **Figure 3D**) and longer event-free survival time (log-rank test, *p*<0.0001; **Supplementary Figure S6B**). The differences in survival curves between the high and low pyroscore patients were also verified in small cohorts (**Supplementary Figures S6C–J**). GSEA results revealed that tumors in the high pyroscore group had gene expressions significantly enriched in the pyroptosis pathway and many immune-related pathways such as adaptive immune response, antigen processing and presentation, and interferon-gamma signaling pathways (**Supplementary Figure 3E**, **Supplementary Table S5**), whereas the low pyroscore tumors had gene expressions enriched in cell cycle and metabolism-related pathways like E2F targets, MYC targets, G2/M checkpoint, oxidative phosphorylation, and MTORC1 signaling pathways (**Figure 3F**). We also examined the mutation landscapes between patients with low and high pyroscores (**Figures 3G–H**). Among the genes that had different mutation frequencies between NB samples scored high for pyroptosis and those scored low, *ALK* stood out in that its mutation frequency was much lower in the high pyroscore samples (**Supplementary Figure S6K**). Since *ALK* gene mutation has been proven to be a susceptibility factor and an important molecular driver for NB (36), the fact that *ALK* mutation frequency negatively correlated with pyroscore further increased our confidence in the efficacy of pyroscore as a marker for prognosis in NB patients.

### Pyroscore Is Associated With Tumor Immune Features

**Figure 4:**
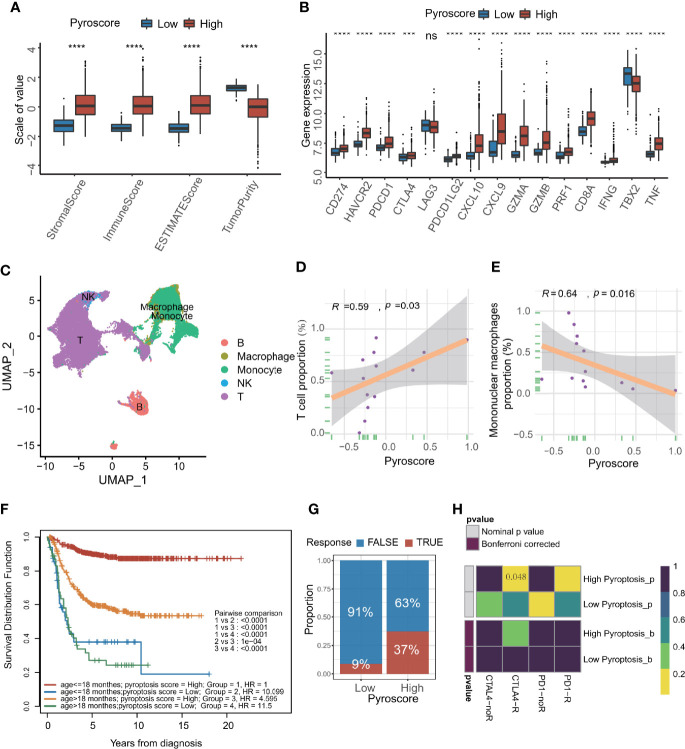
Pyroscore is associated with tumor immune features. **(A)** The value of ESTIMATE score in high and low pyroscore groups (Kruskal–Wallis test, ****p<0.0001). **(B)** Boxplot of immune activate genes and immune checkpoint genes between high and low pyroscore groups (Kruskal–Wallis test, ***p < 0.001; ****p < 0.0001; ns, not significant). **(C)** UMAP plot of the major lineages of tumor-infiltrating lymphocytes (TILs) in NB. **(D)** Scatterplot showing the Spearman correlation of the proportion of T cells and the pyroscore in tumor tissues. **(E)** Scatterplot showed the Spearman correlation of the proportion of mononuclear macrophages and the pyroscore in tumor tissues. **(F)** Kaplan–Meier curves for patients stratified by both age grouping and pyroscore. (log-rank test, *p* < 0.0001, high pyroscore age >18 months versus age ≤18 months; log-rank test, *p* < 0.0001, low pyroscore age>18 months versus age ≤ 18 months). **(G)** Rate of clinical response estimated by TIDE algorithm (response and no response) in high or low pyroscore groups. **(H)** Heatmap visualized the response to anti-CTLA4 and anti-PD-1 therapies between the two groups.

Since the above results showed that pyroscore and immunity seemed to be highly correlated, we further explored the relationship between them. First, through the ESTIMAT algorithm, it was concluded that the immune score in the high pyroscore group was significantly higher than that in the low pyroscore group, but the tumor purity in the high pyroscore group was lower than that in the other group (**Figure 4A**). Then, we evaluated the expression levels of immune checkpoints and immune-activity-related genes. The results revealed that most of these genes were expressed relatively higher in the high pyroscore group (**Figure 4B**). We also estimated the immune cell infiltration both in high and low pyroscore groups, respectively, by the CIBERSORT and ssGSEA algorithm. The proportions of CD8^+^ T cells, CD4^+^ T cells, and activated NK cells in the high pyroscore group were higher than those in the other group (**Supplementary Figures S7A, B**). More tumor immune characteristics were evaluated by the Tumor Immune Dysfunction and Exclusion (TIDE) algorithm (**Supplementary Figure S7C**). The results showed that the pyroscore negatively correlated with suppressive immune cells (M2 TAM and DMSC) and exclusion score, which is a score to evaluate the degree of excluding T cells from infiltrating tumors (**Supplementary Figure S7D–F**). In order to assess tumor-infiltrating lymphocytes more accurately, we analyzed the single-cell RNA-seq data of NB from dataset GSE137804 (**Figure 4C**; **Supplementary Figure S7G**). The results further confirmed that the pyroscore of NB patients was positively correlated with the proportion of T cells and negatively correlated with the proportion of mononuclear macrophages (**Figures 4D, E**).

Patients under 18 months of age are more likely to experience spontaneous regression of tumors, which may be due to activation of immune system (11). The survival analysis of age groups and pyroscore groups revealed that the survival advantage of the high pyroscore group was independent of age grouping by 18 months (children under 18 months are more likely to have a better outcome than older children) (**Figure 4F**).

In order to quantify the response rate of patients to immunotherapy, we examined the prediction results of TIDE algorithm for immunotherapy response in more details. The TIDE algorithm can predict a patient’s response to immunotherapy by computing several published markers based on the expression profile before tumor treatment. The response of each patient to immunotherapy (response or not response) can be evaluated. As expected, a higher pyroscore was associated with better response to cancer immunotherapy (two-sided Fisher’s test, *p*=0.01536; **Figure 4G**; **Supplementary Figure S7H**). By comparing the similarity with the expression profile of a cohort that received immunotherapy, the patients in the high pyroscore group were obviously linked with the patients who were responsive to anti-CTLA4 therapy (*p* = 0.048, **Figure 4H**), which suggests that pyroscore may be used to predict a patient’s response to immunotherapy, especially immune checkpoint inhibitors like anti-CTLA4.

### Pyroscore Is an Independent Prognostic Factor for NB

**Figure 5:**
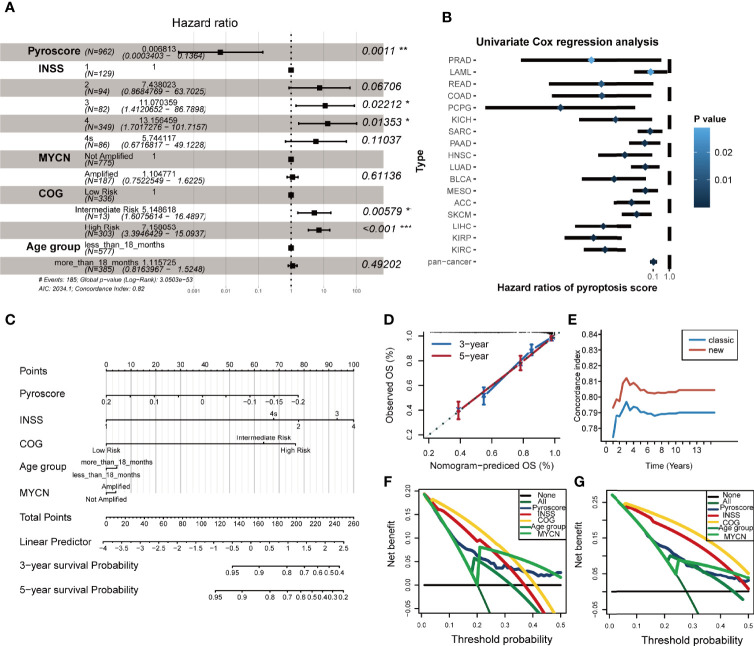
Pyroscore is an independent prognostic factor for NB. **(A)** Forest plot summary of the multivariable Cox analysis of the pyroscore and clinicopathological characteristics (*p < 0.05; **p < 0.01; ***p < 0.001). **(B)** Forest plot showed the univariate Cox analysis of the pyroscore in the TCGA pan-cancer analysis. **(C)** Nomogram plot for predicting the probability of patient mortality at 3- or 5-year OS. **(D)** Calibration curves of the nomogram for predicting the probability of OS at 3 and 5 years. **(E)** Time-dependent c-index of the nomogram. **(F, G)** Decision curve analyses (DCAs) of the nomograms based on five prognosis factors for 3- and 5-year risk.

Given that the pyroscore is related to the clinical and immune characteristics, we tried to determine its role in serving as an independent prognostic factor for NB. First, univariate Cox hazard analysis was applied to the pyroscore, INSS, COG, *MYCN* amplifications, sex, and age grouping (**Supplementary Figure S8A**). The prognostic factors with *p*-value <0.05 in univariate Cox analysis were included in multivariate Cox regression analysis. The results of multivariate Cox regression indicated that the pyroscore was a significant protective factor for NB (**Figure 5A**). Furthermore, a total of 33 cancers (**Supplementary Table S6**) from The Cancer Genome Atlas (TCGA) database were also used to evaluate the validity and universality of the pyroscore. The pyroscore had a significant effect on prognosis in 17 of the cancers, and in all of them, the pyroscore performed as a protective factor (**Figure 5B**).

In addition, we built two models to assess the benefit of pyroscore to predict prognosis in NB. The classic model included the variables that are currently recognized and had significant differences in univariate Cox analysis (INSS, COG, *MYCN* amplifications, and age grouping), whereas the new model included the pyroscore besides the four factors used in classic model. Net reclassification index (NRI) was used to measure the improvement of the new model in predictive efficacy. The 3- and 5-year NRI were 0.224 and 0.148, respectively, meaning that the proportion of the new model that had been properly classified increased by 22.4% and 14.8%. The overall improvement of the new model was determined by Integrated Discrimination Improvement (IDI), and the results showed that the IDI>0 and *p*<0.05 (IDI=0.012, *p*=0.016), which suggests that the new model prediction was more accurate when the pyroscore was added. At the same time, the receiver operating characteristic (ROC) curves were executed, and the time-dependent area under the curve of ROC (time-dependent AUC) values of the two models (**Supplementary Figures S8B, C**) confirmed that the new model was a better one.

In order to make the new model (pyroscore, INSS, COG, *MYCN* amplifications, sex, and age grouping) more widely applicable to clinical practice, we constructed a nomogram plot to predict the survival rate of NB patients (**Figure 5C**). Each patient was assigned a comprehensive score, which was calculated by adding the corresponding scores for each variable in the nomogram plot. The accuracy and clinical usefulness of the comprehensive score and the new model were determined by 3- and 5-year calibration curves (**Figure 5D**). Time-dependent C-index curves with more time points also showed better performances in the new model (**Figure 5E**). Decision curve analyses (DCAs) of 3- and 5-year showed the net benefit provided by each prediction factor (**Figures 5F, G**), with pyroscore being a good one.

### Prediction of Drug Sensitivity in Pyroptosis Groups

**Figure 6:**
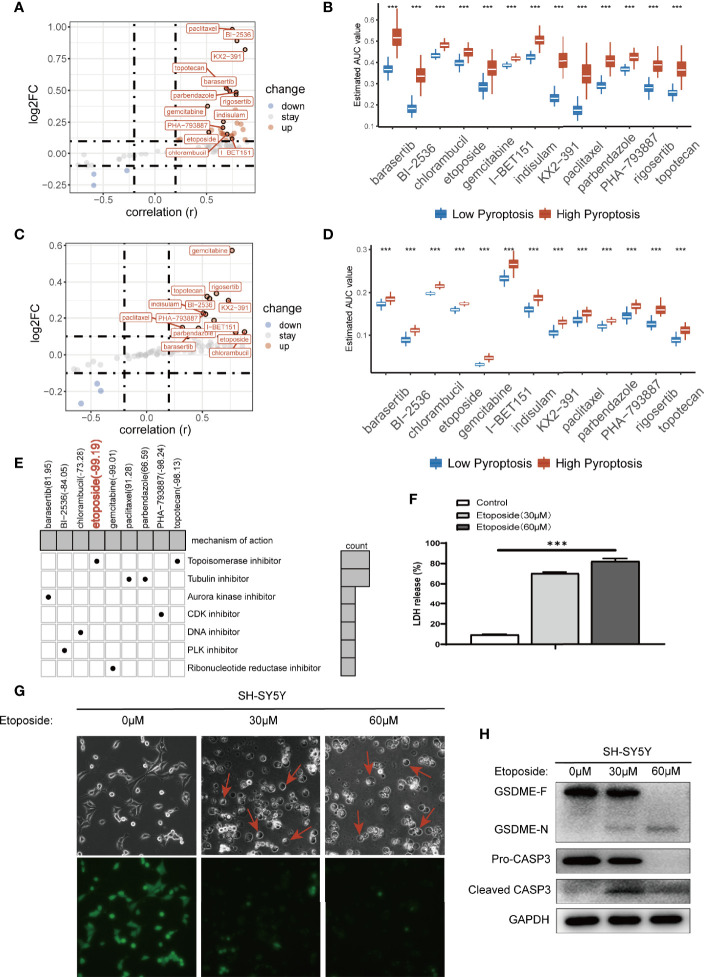
Prediction of drug sensitivity in pyroptosis groups. **(A)** Scatter plot of drug prediction in CTRP database. The x-axis indicates the correlation; the y-axis indicates log_2_FC. **(B)** The results of differential drug response analysis of candidate drugs in CTRP database (Wilcoxon test, ***p < 0.001). **(C)** Scatter plot of drug prediction in PRISM database. The x-axis indicates the correlation; the y-axis indicates log_2_FC. **(D)** The results of differential drug response analysis of candidate drugs in PRISM database (Wilcoxon test, ***p < 0.001). **(E)** Heatmap showed CMap scores of candidate drugs and drug mechanisms of action (rows) through the CMap database. **(F)** LDH release-based cell death assay (data shown as mean ± SD from three technical replicates) in SH-SY5Y cells with 30 μm/60 μm etoposide (***p < 0.001). **(G)** Images of etoposide induced pyroptosis in SH-SY5Y cells. Calcein-AM staining was used for the cell death assay; green indicates living cells. **(H)** Western blot analysis of proCaspase-3, cleaved caspase-3, and GSDME in SH-SY5Y cells in 48 h after treating with 30 μm/60 μm etoposide.

Next, we tried to find drugs that were sensitive to the pyroptosis phenotype using the pyroscore model to guide clinical medication for NB patients. We first used two drug sensitivity databases (CTRP and PRISM) to identify drug candidates based on AUC values. CTRP includes 299 drugs, and PRISM includes 1,285 drugs. A total of 78 drugs after the intersection of the two databases were selected and analyzed. By correlation and multiple variation analysis with thresholds as r>0.2 and log_2_FC>0.1, 13 drugs that overlapped in the two databases were selected at first (**Figures 6A, C**), and all of them had lower AUC values in the low pyroscore group of both databases (**Figures 6B, D**). We further utilized CMap to determine the performance of drug candidates. The CMap score inversely correlates with the potential therapeutic effect of a drug. The CMap values of six drugs were <0, indicating their potential ability of inducing pyroptosis in the low pyroscore group, and the therapeutic targets of these drugs were shown as well (**Figure 6E**). The above results predict that etoposide may be the most promising drug to induce pyroptosis in NB, especially that it has the lowest CMap score. To verify the predicted results, we treated human NB cell line SH-SY5Y with etoposide and successfully induced pyroptosis. The proportion of LDH release (**Figure 6F**) increased significantly after etoposide treatment. Cells treated with etoposide exhibited large bubbles emerging from plasma membrane and cell swelling (**Figure 6G**). Calcein-AM staining assay also demonstrated that etoposide induced cell death (**Figure 6G**). The cleavage of GSDME and the activation of CASP3 were simultaneously detected in cells treated with etoposide by Western blot assay (**Figure 6H**). Therefore, etoposide, which was predicted by our model that had the ability of inducing pyroptosis in NB with low pyroscore, could induce pyroptosis *in vitro*.

### The Pyroscore Predicts Immunotherapeutic Benefits

**Figure 7:**
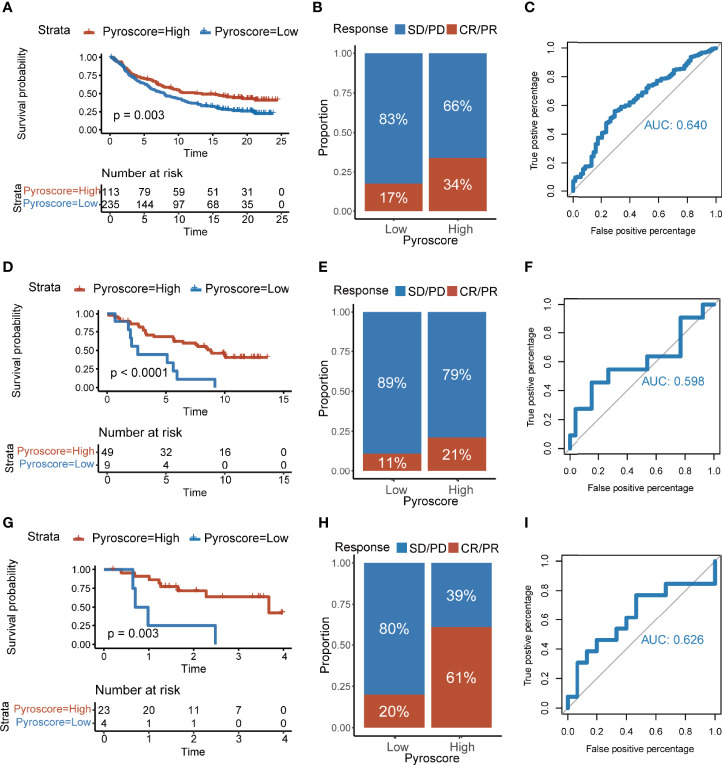
The pyroscore predicts immunotherapeutic benefits. **(A)** Kaplan–Meier curves for patients with high and low pyroscore in the IMvigor210 cohort (log-rank test, *p*=0.003). **(B)** Rate of clinical response [complete response (CR), partial response (PR), stable disease (SD), progressive disease (PD)] to immunotherapy in high or low pyroscore groups in the IMvigor210 cohort. **(C)** ROC curve of the pyroscore in the IMvigor210 cohort. **(D)** Kaplan–Meier curves for patients with high and low pyroscore in the GSE91061 cohort (log-rank test, *p*< 0.001). **(E)** Rate of clinical response (CR and PR with SD and PD) to immunotherapy in high or low pyroscore groups in the GSE91061 cohort. **(F)** ROC curve of the pyroscore in the GSE91061 cohort. **(G)** Kaplan–Meier curves for patients with high and low pyroscore in the GSE78220 cohort (log-rank test, *p*=0.003). **(H)** Rate of clinical response (CR and PR with SD and PD) to immunotherapy in high or low pyroscore groups in the GSE78220 cohort. **(I)** ROC curve of the pyroscore in the GSE78220 cohort.

ICB therapy has shown exciting clinical outcomes in cancers, and our previous results showed robust evidence that the patients of high pyroscore were more likely to benefit from ICB therapies. We decided to further explore the relationship between the pyroscore and the benefit of ICB therapy using external cohorts. A total of four cohorts that received immune checkpoint treatments were collected: IMvigor210, GSE91061, GSE78220, and GSE35640. It was demonstrated in IMvigor210 (anti-PD-L1) cohort that a higher pyroscore was associated with a significantly better prognosis (log-rank test, *p*=0.003) and better response to anti-PD-L1 therapy (two-sided Fisher’s exact test, *p*=0.015), indicating that the patients of high pyroscore were more likely to benefit from ICB therapy (**Figures 7A, B**). ROC curve also evaluated and proved the accuracy of pyroscore in predicting responsiveness to ICB therapy (**Figure 7C**, AUC=0.640). The same conclusions were found in GSE91061 (anti-PD-1) and GSE78220 (anti-PD-1) cohorts (**Figures 7D–I**). In addition, the pyroscore was associated with immune-infiltrating phenotype in the IMvigor210 cohort (**Supplementary Figure S9A**). Finally, in the GSE35640 (anti-MAGE-A3) cohort, the pyroscore was significantly higher in the group that responded to ICB therapy (**Supplementary Figures S9B, C**). Similarly, ROC curve showed that pyroscore had a good predictability for immunotherapy benefits (**Supplementary Figure S9D**). Taken together, our data strongly suggested that the pyroscore could be a good predictor of response to immune checkpoint blockade treatments.

## Discussion

Pyroptosis that was initially studied in macrophages was redefined in 2017 (37). It has been found that pyroptosis is closely related to some diseases like autoimmune diseases and hearing loss (38, 39). Recently, more and more reports have studied the role of pyroptosis in cancers, but the conclusions are not consistent. Pyroptosis may play an anti- or pro-tumor roles in different cancers depending on cellular background (15, 40). On the one hand, the inflammatory mediators released during pyroptosis are favorable for tumor cell growth and thus promote the development of tumors. On the other hand, pyroptosis as a type of cell death can inhibit the occurrence and growth of tumor cells. Thus, the role of pyroptosis in tumor deserves further study. Recent studies have demonstrated that induction of pyroptosis in malignant cells could kill cancer cells and may function as a tumor suppressor (41). Many studies indicate that therapeutic regimens such as chemotherapy, radiotherapy and immunotherapy could trigger pyroptosis in tumor, which further potentiate its anti-tumor immunity (42, 43). In this study, we determined the cell non-autonomous tumor suppressor effect of pyroptosis in NB. We proposed that pyroptosis of NB cells can trigger an immune response and is related to the benefit of immunotherapy.

In this study, consensus clustering analysis of pyroptosis genes was used to determine three pyroptosis groups, which were different in survival time and immune cell infiltration. To further explore the underlying molecular mechanisms of different pyroptosis groups, we obtained differentially expressed genes among the three groups. Three patient clusters were defined by consensus clustering of differentially expressed genes. The cluster of patients with the worst overall survival time had a higher proportion of suppressive immune cells such as macrophages and Tregs. Finally, non-redundant biomarkers were determined through the Boruta algorithm and used to construct a pyroscore.

The complex gene network needed to be quantified by a single indicator. Principal components analysis is a reliable machine learning method that can reduce the dimensionality of data through linear transformation and has been widely used for biological quantification (44). Through the PCA algorithm, we established a novel scoring method called pyroscore to quantify the pyroptosis subtypes. GSEA results showed that genes in the high pyroscore group were significantly enriched in the immune-related pathways for active immune environment, such as T-cell activation, adaptive immune response, and antigen processing and presentation. Whereas in the low pyroscore group, genes were enriched in the DNA replication and glycolysis pathways, which were generally considered to indicate a poor prognosis (45, 46) because increased DNA replication and glycolysis by tumors may raise immune resistance (47). The pyroscore was also significantly related to the clinicopathological characteristics of NB patients. The NB staging factoring currently includes INSS, COG, *MYCN* amplification, and age grouping. The pyroscore that we constructed inversely correlates with INSS, COG, *MYCN* amplification, and age grouping and is independent of sex, suggesting it is a protective factor for NB. When the pyroscore was included into the traditionally used model (INSS, COG, *MYCN* amplification, and age grouping), the new model showed a better predict accuracy for prognosis.

The relationship between pyroscore and tumor immunity was evaluated from multiple perspectives. High pyroscore was associated with high immune score, low tumor purity, and high infiltration of CD8^+^ T cells/CD4^+^ T cells/resting dendritic cells, which indicates that pyroptosis is associated with active immune environment. The results from our analysis are consistent with several recent published studies showing that pyroptosis may induce antitumor immunity and reduce tumor load in mouse models (15, 41, 48). It is known that NB patients in different age groups exhibit different immune status (11). Survival analysis of age grouping and pyroscore groups revealed in this study that pyroscore was a good prognostic factor independent of age, which has long been used as a prognostic factor because NB patients with age ≤18 months are associated with good prognosis. Therefore, pyroptosis is another protective factor for NB besides age ≤18 months.

Comprehensive and systematic clinical model evaluation indicators were used in this study to assess the pyroscore, which has been proven to have good clinical effect. The pyroscore serves as an independent prognostic factor in NB and could accurately predict the survival status of NB patients. The new model, which incorporated the pyroscore, performed better predictive effect than the classic model built by INSS, COG, *MYCN* amplification, and age grouping.

We screened out etoposide, a topoisomerase II inhibitor, which had the lowest CMap score based on the two drug sensitivity databases. A great number of studies have demonstrated that etoposide can induce apoptosis rather than pyroptosis, and etoposide treatment did not activate CASP1 in bone-marrow-derived macrophages (49, 50). However, some literatures report that etoposide can induce cells change from apoptosis to pyroptosis (42, 51). It is interesting that we found that etoposide had the ability of inducing pyroptosis in NB.

TIDE and Submap algorithms predicted that patients in the group with high pyroscore had a better response to ICB therapy. Four cohorts that had received ICB therapy treatments were used to evaluate and verify the predictive value of the pyroscore. Consistent with previous studies (52) and with our prediction, levels of pyroscore were associated with the expression of immune checkpoint genes. Published clinical trials have shown that PD-L1 inhibitors combined with chemotherapy can kill cancer cells by triggering the pyroptosis of cancer cells. This may improve the survival of patients and increase the efficiency of PD-L1 inhibitors (53). We observed a significantly higher pyroscore in responders than in non-responders undergoing ICB therapy, indicating that single-agent immunotherapy might be beneficial for the patients with high pyroscore.

This study has produced some insights into the comprehensive assessment of cellular and molecular factors related to pyroptosis, revealing that tumor pyroptosis triggers a gene network associated with active immune response and responds to immunotherapy, and may help clinical practitioners choose to appropriate treatment plan and predict prognosis for NB patients.

## Data Availability Statement

The original contributions presented in the study are included in the article/**Supplementary Material**. Further inquiries can be directed to the corresponding authors.

## Author Contributions

BL designed the study, analyzed the data, and wrote the manuscript. LW performed the literature search and collected data for the manuscript. WG conducted the experiment. YS, YL, JZ, JY, QZ, JL, JY, QZ, and YD provided resources. LL conceived and designed the experiments, interpreted the results, wrote and revised this manuscript. All authors contributed to the article and approved the submitted version.

## Funding

This work was supported by funding from the National Key Research and Development Program of China (Nos. 2018YFC1313000 and 2018YFC1313002).

## Conflict of Interest

The authors declare that the research was conducted in the absence of any commercial or financial relationships that could be construed as a potential conflict of interest.

## Publisher’s Note

All claims expressed in this article are solely those of the authors and do not necessarily represent those of their affiliated organizations, or those of the publisher, the editors and the reviewers. Any product that may be evaluated in this article, or claim that may be made by its manufacturer, is not guaranteed or endorsed by the publisher.

## Abbreviations

NB, neuroblastoma; ICB, immune checkpoint blockade; TME, tumor microenvironment; CAFs, cancer associated fibroblasts; MSCs, mesenchymal stromal cells; FPKM, transcript per million fragments mapped; TPM, transcripts per million; PAC, proportion of ambiguous clustering; DEG, differentially expressed gene; PCA, principal component analysis; ORA, over representation analysis; GO, gene ontology; GSEA, gene set enrichment analysis; GSVA, gene set variation analysis; CMap, connectivity map; TIDE, tumor immune dysfunction and exclusion; UMAP, uniform manifold approximation and projection; INSS, international neuroblastoma staging system; COG, Children’s Oncology Group; NRI, net reclassification index; IDI, integrated discrimination improvement, ROC, receiver operating characteristic; AUC, area under the curve of ROC; DCA, decision curve analyses.
