# Supplementary material for: Using a Gene Network of Pyroptosis to Quantify the Responses to Immunotherapy and Prognosis for Neuroblastoma Patients
Source: Front Immunol. 2022 Mar 24;13:845757. doi: 10.3389/fimmu.2022.845757 (PMC8987018; doi:10.3389/fimmu.2022.845757)
Supplement: Supplementary file 1 [file DataSheet_1.pdf]

Supplementary Material

This PDF file includes:

Figs S1 to S9

Tables S1 to S6

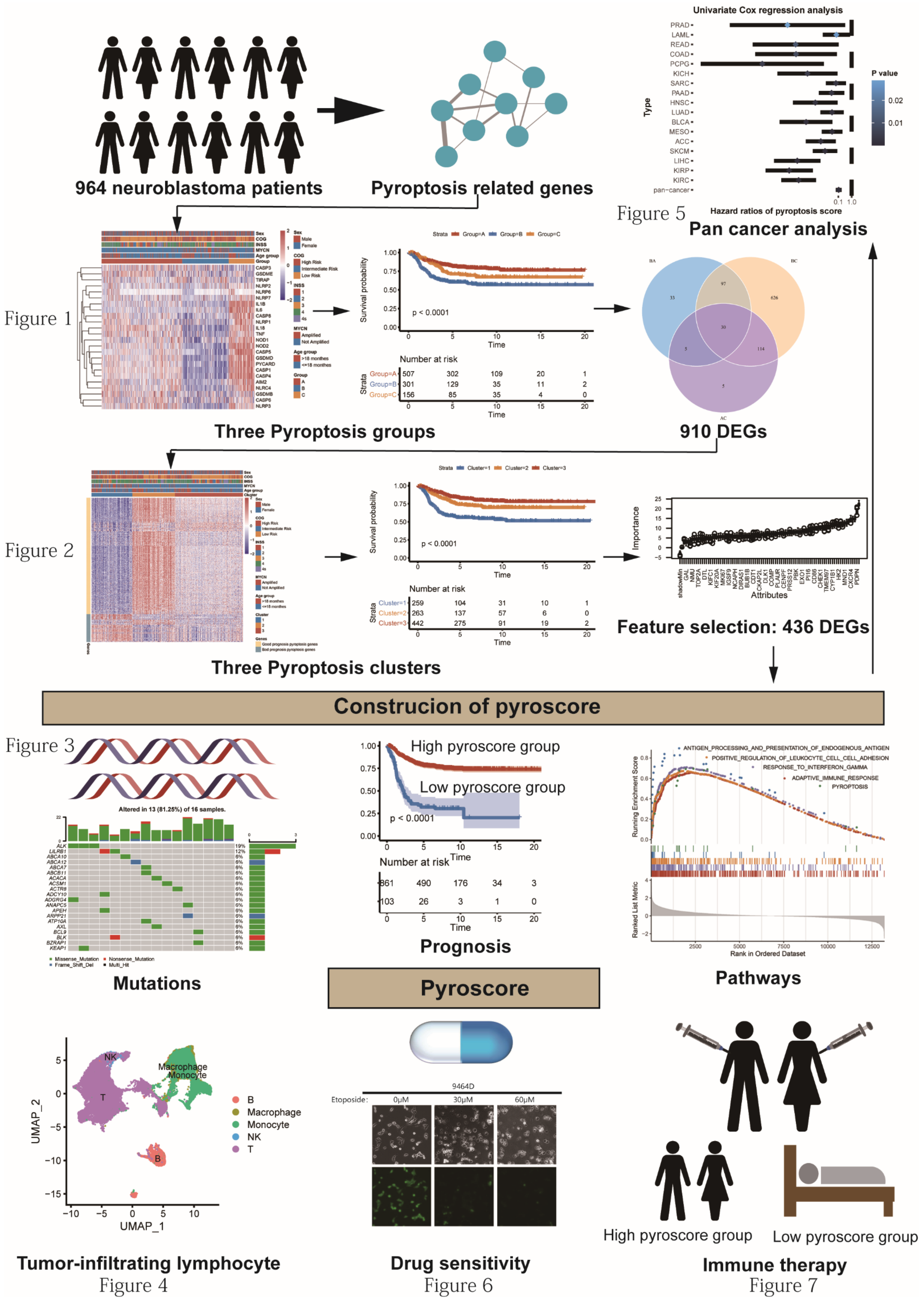

Figure S1. Workflow of this project.

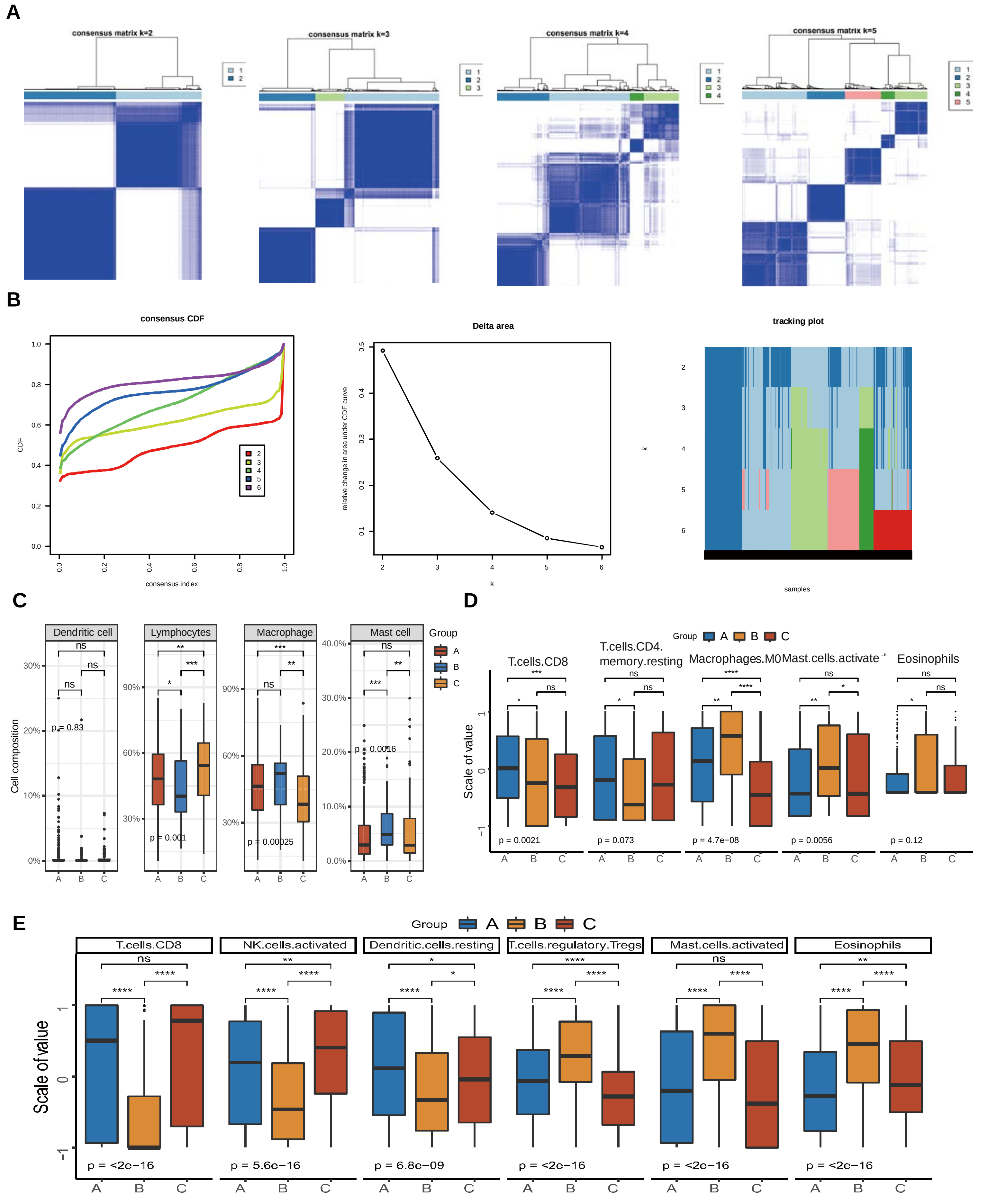

Figure S2. Pyroptosis groups of 964 NB patients. (A) Consensus matrixes of all meta-NB cohorts for each k (k = 2-5). (B) Outputs of consensus clustering: The Cumulative Distribution Function (CDF) Plot on the left, Delta Area Plot in the middle, and Tracking Plot on the right. (C) Boxplot of Immune cell infiltration in three pyroptosis groups. The results of the CIBERSORT algorithm were classified into four types of immune cells: total lymphocytes, total dendritic cell, total macrophage and total mast cell. (D) Boxplot of Immune cell infiltration inferred by CIBERSORT in three pyroptosis groups. (E) Boxplot of Immune cell infiltration inferred by ssGSEA in three pyroptosis groups.

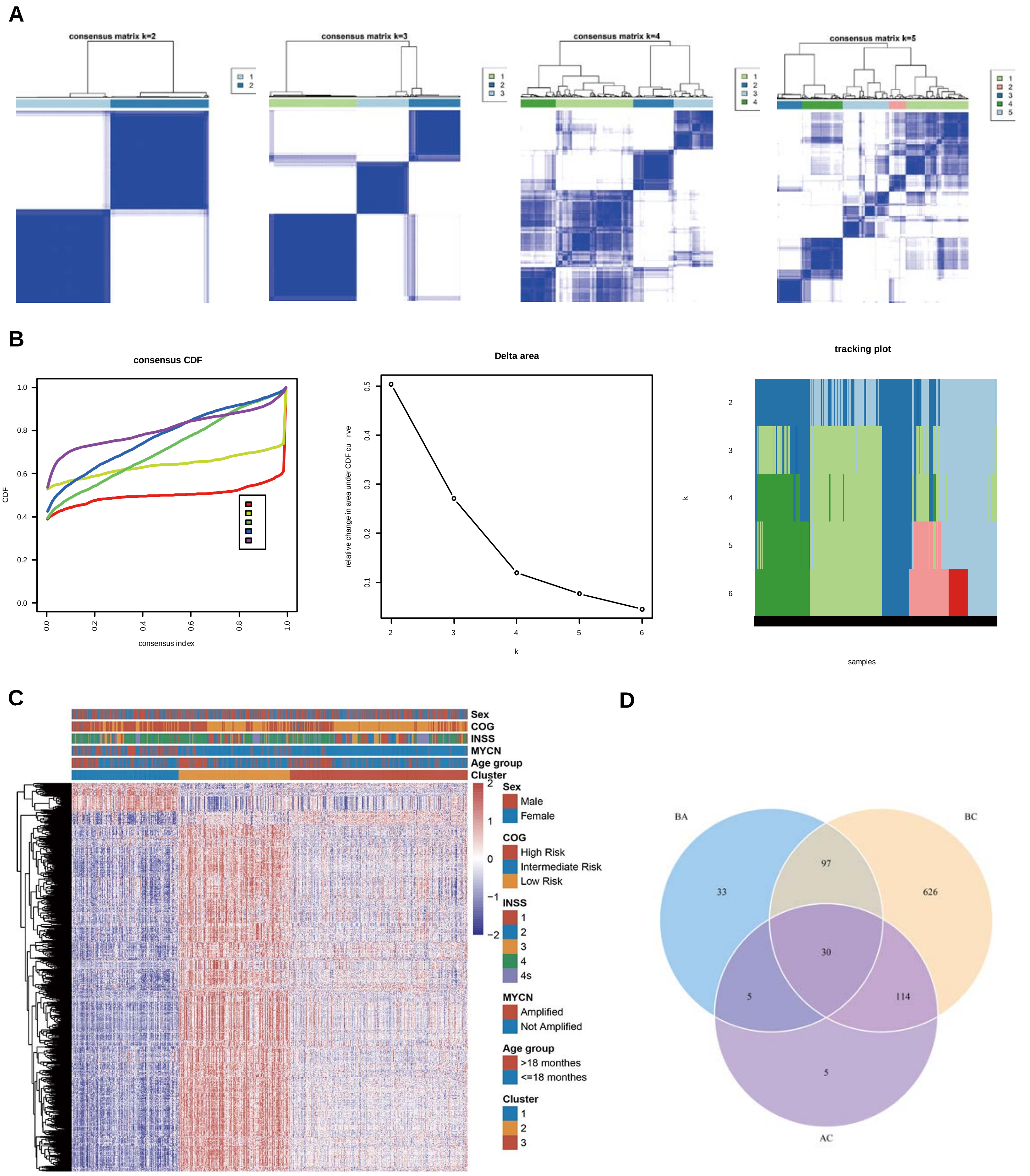

Figure S3. Consensus clustering of DEGs among the pyroptosis groups. (A) Consensus matrixes of all meta-NB cohorts for each k (k = 2–5). (B) Outputs of consensus clustering: The Cumulative Distribution Function (CDF) Plot on the left, Delta Area Plot in the middle, and Tracking Plot on the right. (C) Unsupervised consensus clustering of 910 DEGs among three pyroptosis cluster groups from the meta-NB cohort. (D) Venn diagram illustrating the number of DEGs among the three pyroptosis groups.

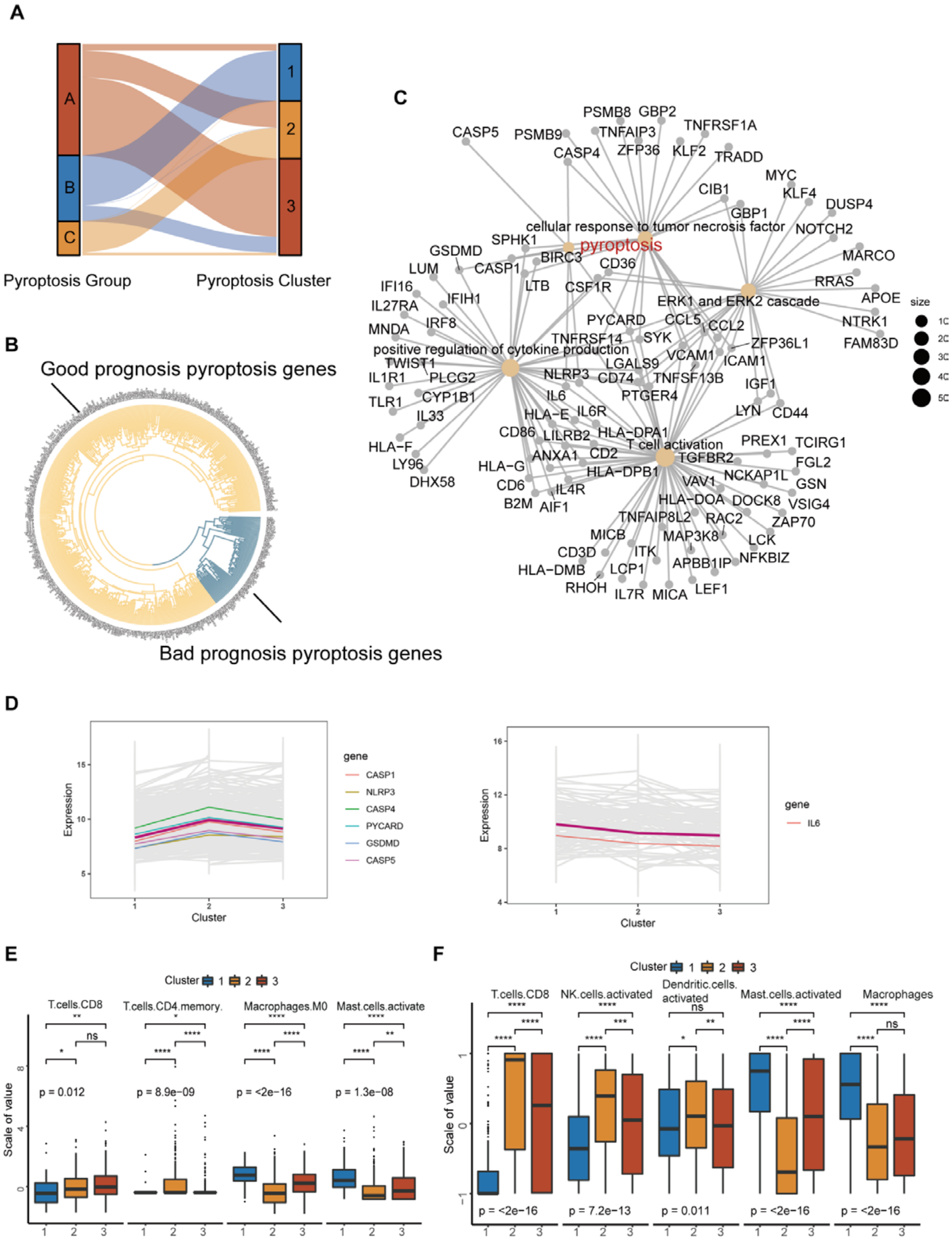
A
Pyroptosis Group
Pyroptosis Cluster
B
Good prognosis pyroptosis genes
Bad prognosis pyroptosis genes
C
pyroptosis
cellular response to tumor necrosis factor
ERK1 and ERK2 cascade
positive regulation of cytokine production
T cell activation
size
D
gene
CASP1
NLRP3
CASP4
PYCARD
GSDMD
CASP5
IL6
Expression
Cluster
E
Cluster 1 2 3
T.cells.CD8
T.cells.CD4.memory.
Macrophages.M0
Mast.cells.activate
p = 0.012
p = 8.9e-09
p = <2e-16
p = 1.3e-08
Scale of value
F
Cluster 1 2 3
T.cells.CD8
NK.cells.activated
Dendritic.cells.
activated
Mast.cells.activated
Macrophages
p = <2e-16
p = 7.2e-13
p = 0.011
p = <2e-16
p = <2e-16
Scale of value

Figure S4. The functional annotation of pyroptosis signature genes and TME in three clusters. (A) Sankey diagram showing differences among patients by groups and clusters. (B) Hierarchical clustering of good prognosis pyroptosis genes (GPPGs, yellow) and bad prognosis pyroptosis genes (BPPGs, blue). (C) Potential regulatory network of pyroptosis signature genes. (D) Clustered line chart for GPPGs and BPPGs. On the left: GPPGs, on the right: BPPGs. (E) Boxplot of Immune cell infiltration inferred by ssGSEA in three pyroptosis clusters. (F) Boxplot of Immune cell infiltration inferred by CIBERSORT in three pyroptosis clusters.

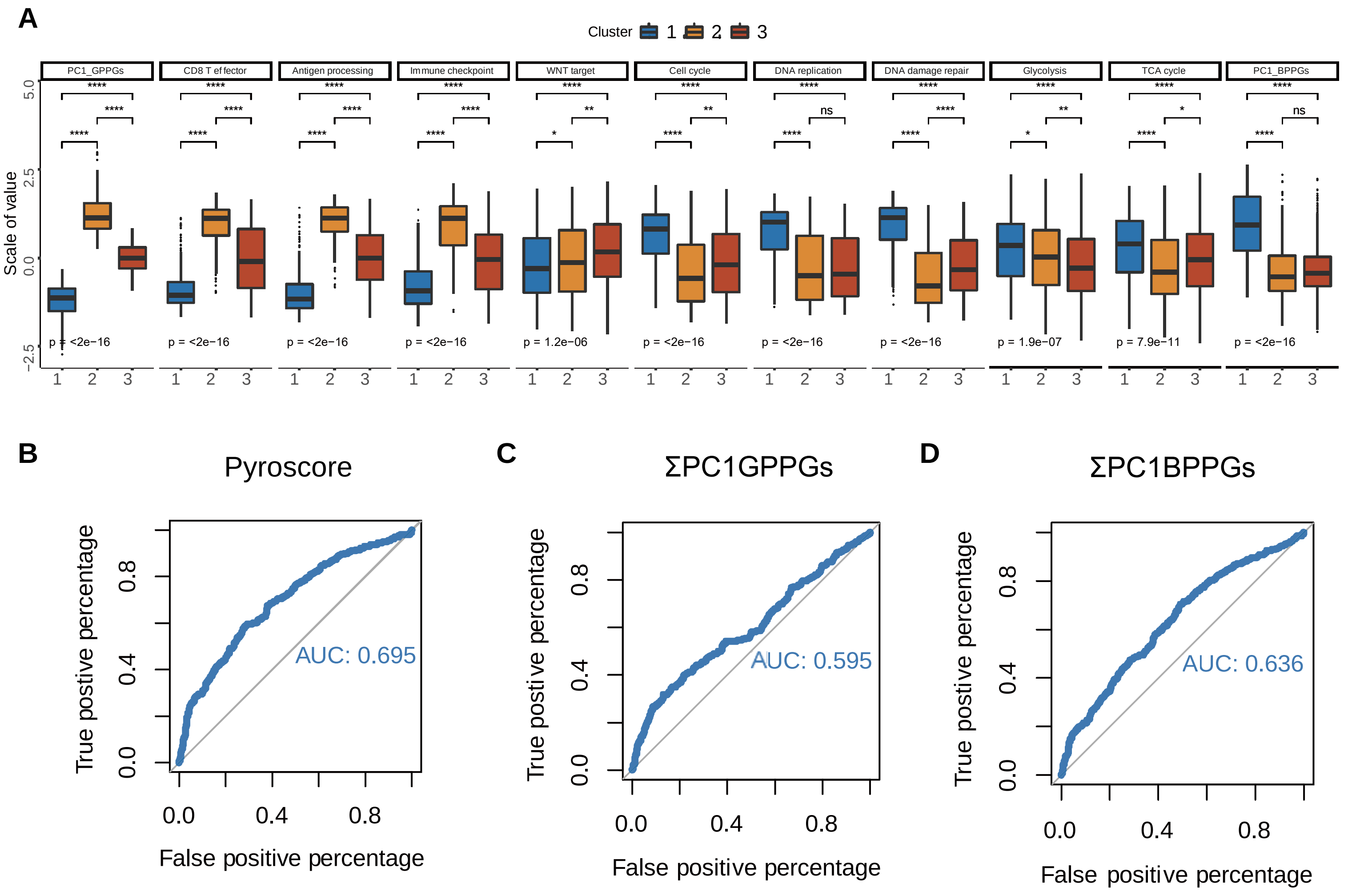

Figure S5. Characteristics of ΣPC1GPPGs and ΣPC1BPPGs. (A) ΣPC1GPPGs and ΣPC1BPPGs are related to different pathways. (B) ROC curve based on pyroscore for each patient (AUC=0.695). (C) ROC curve based on ΣPC1GPPG for each patient (AUC=0.595). (D) ROC curve based on ΣPC1BPPG for each patient (AUC=0.636).

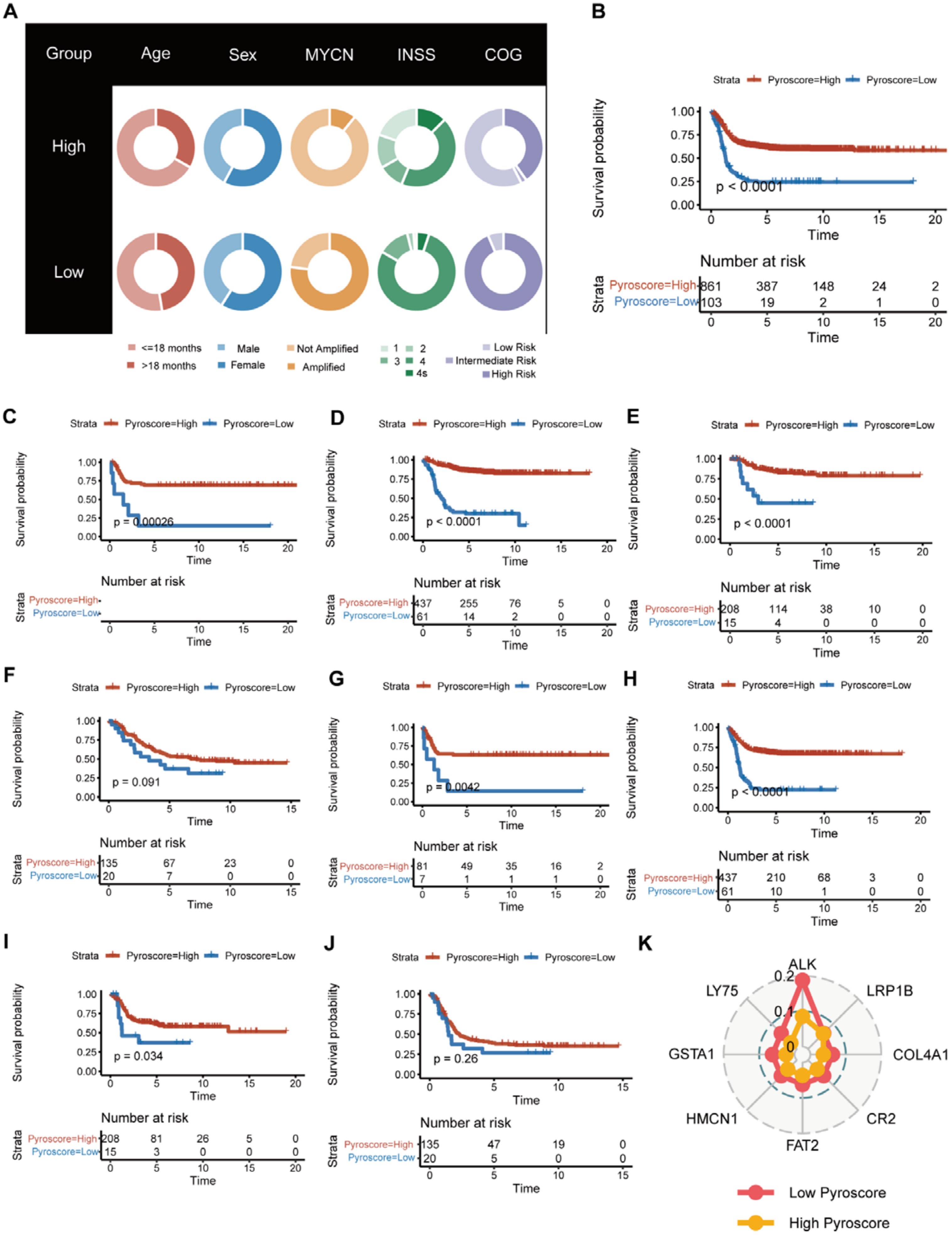
A
Group
Age
Sex
MYCN
INSS
COG
High
Low
<=18 months
>18 months
Male
Female
Not Amplified
Amplified
1
2
3
4
4s
Low Risk
Intermediate Risk
High Risk
B
Strata
Pyroscore=High
Pyroscore=Low
Survival probability
p < 0.0001
Time
Number at risk
Pyroscore=High 861 387 148 24 2
Pyroscore=Low 103 19 2 1 0
C
p = 0.00026
D
p < 0.0001
Pyroscore=High 437 255 76 5 0
Pyroscore=Low 61 14 2 0 0
E
p < 0.0001
Pyroscore=High 208 114 38 10 0
Pyroscore=Low 15 4 0 0 0
F
p = 0.091
Pyroscore=High 135 67 23 0
Pyroscore=Low 20 7 0 0
G
p = 0.0042
Pyroscore=High 81 49 35 16 2
Pyroscore=Low 7 1 1 1 0
H
p < 0.0001
Pyroscore=High 437 210 68 3 0
Pyroscore=Low 61 10 1 0 0
I
p = 0.034
Pyroscore=High 208 81 26 5 0
Pyroscore=Low 15 3 0 0 0
J
p = 0.26
Pyroscore=High 135 47 19 0
Pyroscore=Low 20 5 0 0
K
ALK
LRP1B
COL4A1
CR2
FAT2
HMCN1
GSTA1
LY75
Low Pyroscore
High Pyroscore

Figure S6. Clinicopathological characteristics and prognosis of high and low pyroscore groups. (A) Clinicopathological characteristics (Age grouping, Sex, MYCN, INSS, COG) of high and low pyroscore groups. (B) Kaplan-Meier curves for event free survival time (EFS) of all NB patients with high and low pyroscore groups (log-rank test, $p<0.0001$). (C-F) Kaplan-Meier curves for overall survival (OS) of NB patients with high and low pyroscore groups. From left to right: GSE16476, GSE49710, E-MTAB-8248, TARGET-NB. (G-J) Kaplan-Meier curves for event free survival time (EFS) of NB patients with high and low pyroscore groups. From left to right: GSE16476, GSE49710, E-MTAB-8248, TARGET-NB. (K) Radar chart of gene mutation frequency of the high and low pyroscore groups.

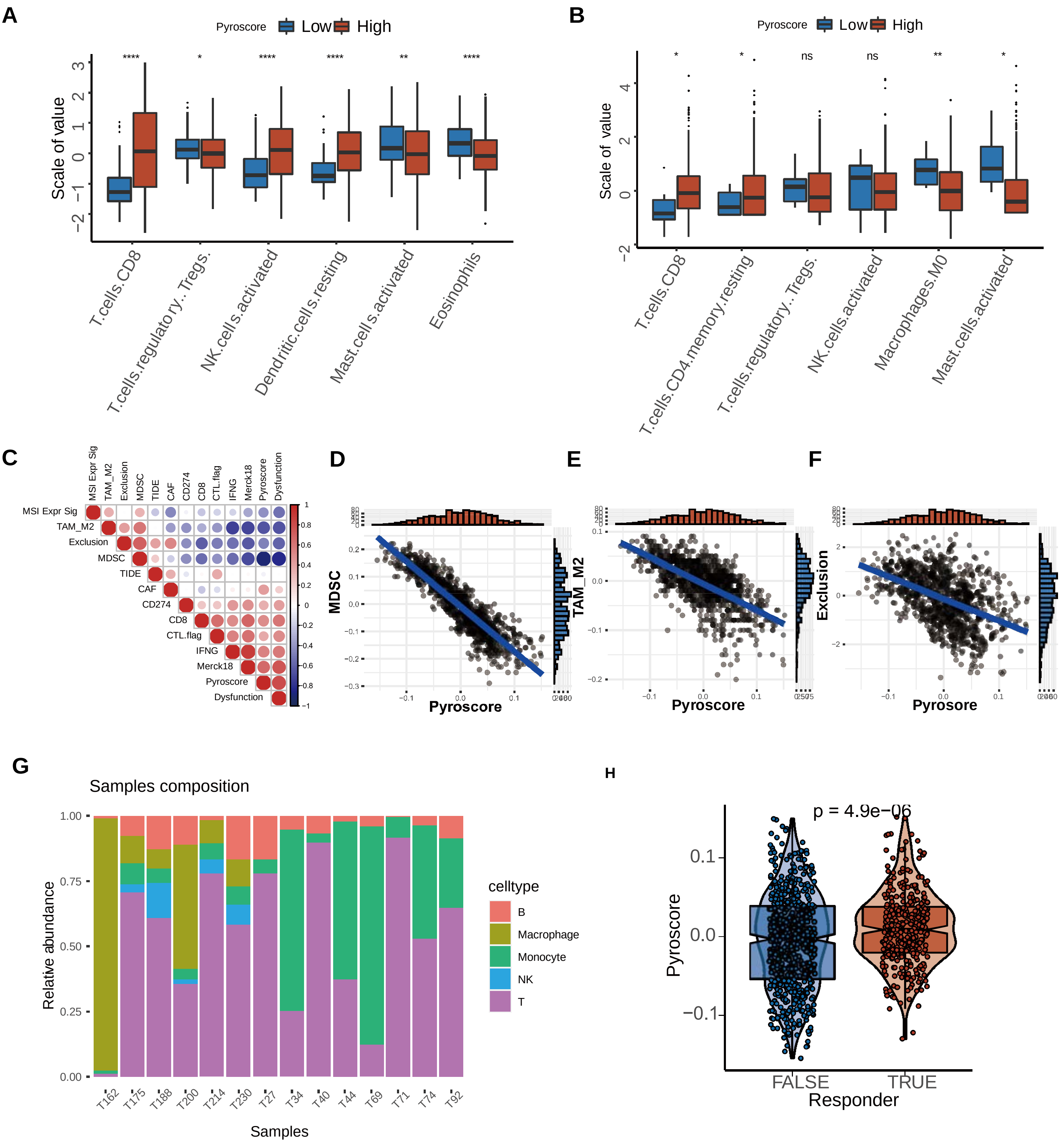

Figure S7. The relationship between the tumor infiltrating lymphocytes and the pyroscore. (A) Boxplot of Immune cell infiltration inferred by ssGSEA in high and low pyroscore subgroups. (B) Boxplot of Immune cell infiltration inferred by CIBERSORT in high and low pyroscore subgroups. (C) Heatmap of the correlation between the results of the TIDE algorithm and the pyroscore. (D) Correlation scatter plot of MDSC score and the pyroscore. (E) Correlation scatter plot of M2 macrophages score and the pyroscore. (F) Correlation scatter plot of exclusion score and the pyroscore. (G) Proportion of tumor infiltrating lymphocytes among patients. (H) Violin plot of pyroscore levels for immunotherapy responsiveness groups (response or no response) predicted by TIDE algorithm.

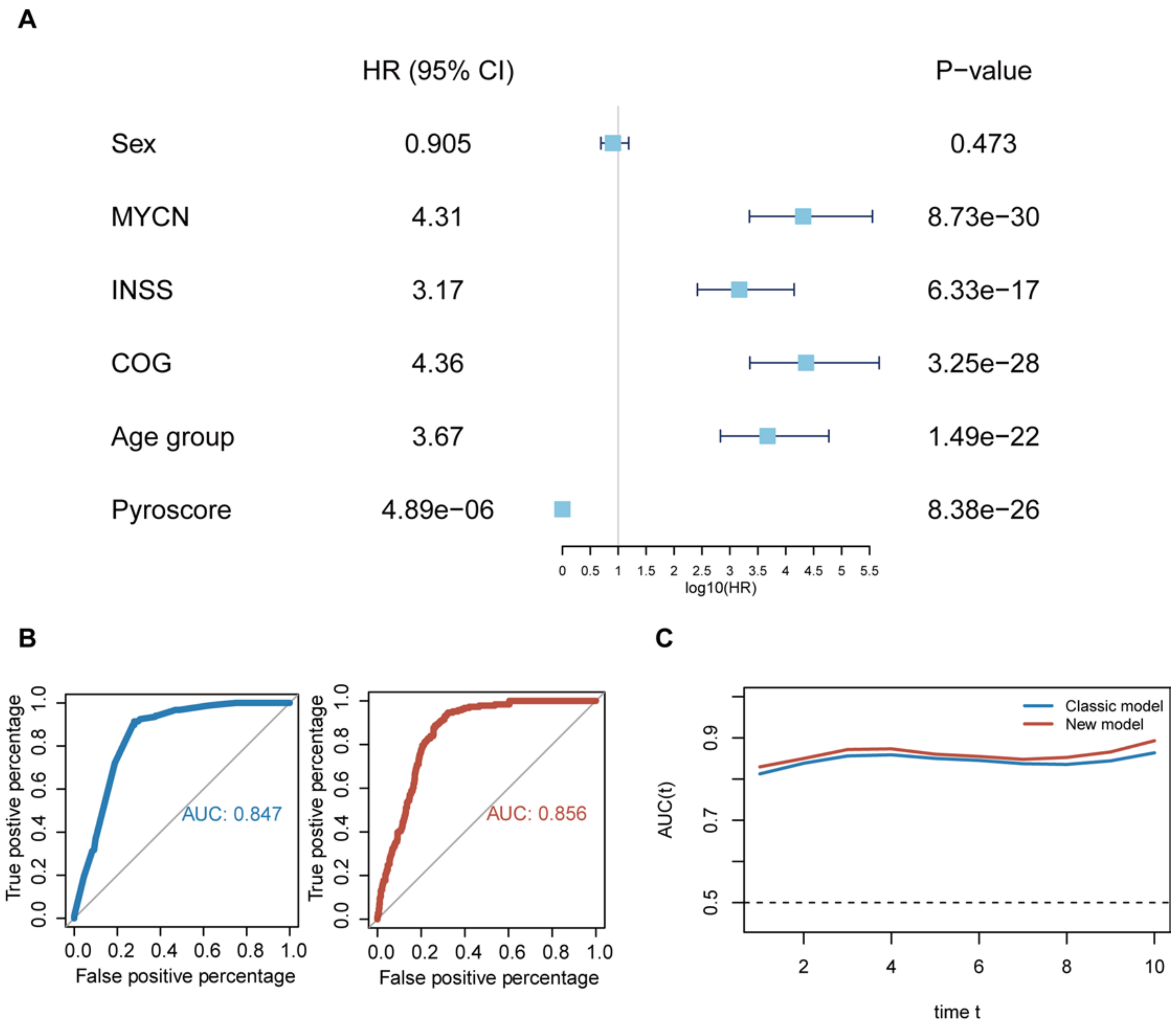

Figure S8. Pyroscore increased clinical prediction model performance. (A) Forest plot showed the univariate Cox analysis of the pyroscore in meta-NB cohort. (B) ROC curves of the two models: classic model (blue, AUC=0.847) and new model (red, AUC=0.856). (C) Time-AUC curves of two models: classic model (blue) and new model (red).

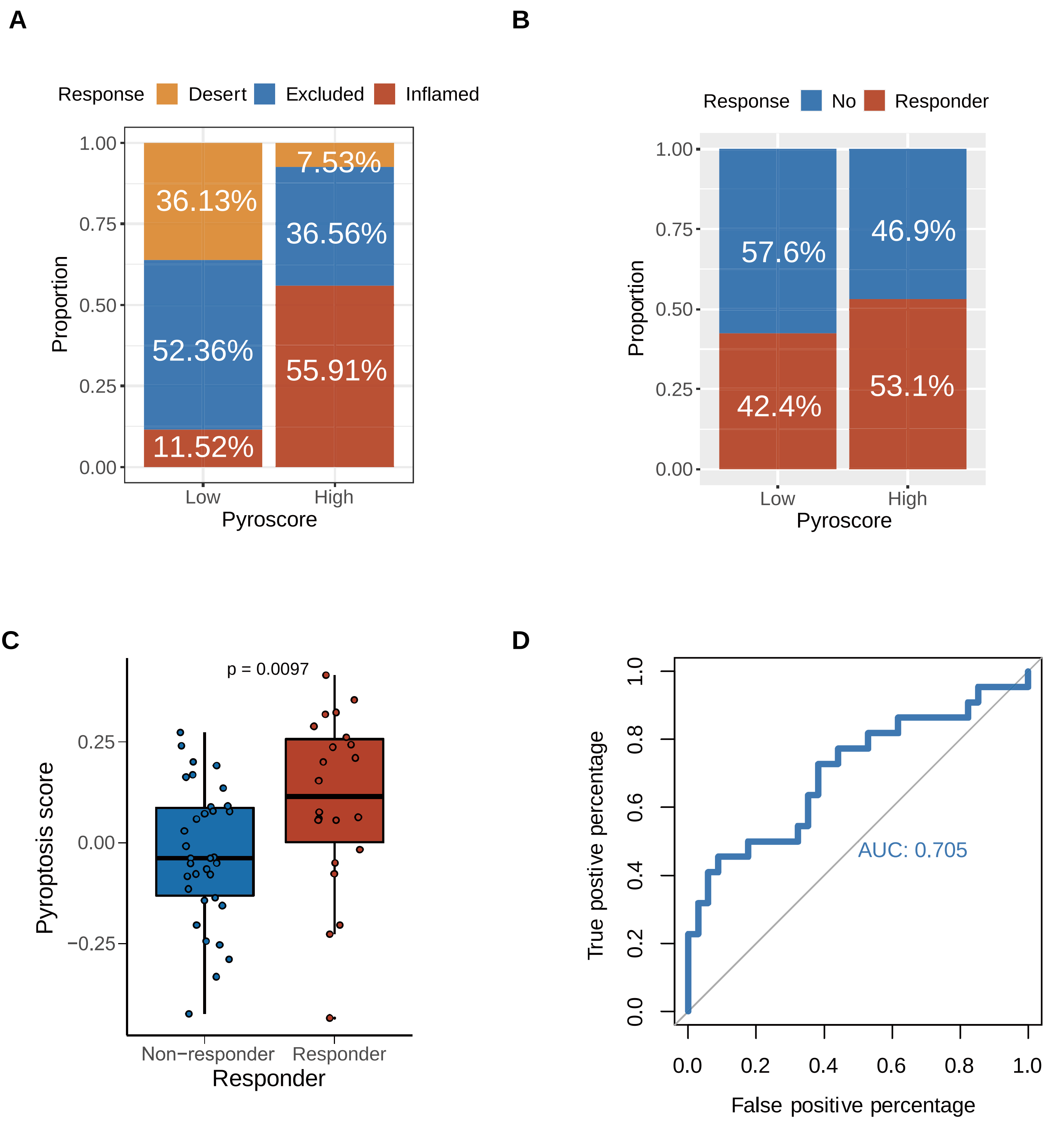

Figure S9. The immune infiltrating phenotypes and predictive value of pyroscore. (A) Rate of immune infiltrating phenotypes (Desert/ Excluded/ Inflamed) to immunotherapy in high or low pyroscore groups in the IMvigor210 cohort. (B) Rate of clinical response (CR/PR and SD/PD) to immunotherapy in high or low pyroscore groups in the GSE35640 cohort. (C) Distributions of pyroscore in different response status to MAGE-A3 immunotherapeutic (GSE35640). (D) ROC curve of the pyroscore in the GSE35640 cohort.

SUPPLEMENTARY TABLES

Table S1. Relative scores of tumor-infiltrating immune stat by ESTIMATE of neuroblastoma patients.

Table S2. Differentially expressed genes of pyroptosis groups in neuroblastoma.

Table S3. Good prognosis pyroptosis genes (GPPGs) and Bad prognosis pyroptosis genes (BPPGs).

Table S4. Gene ontology (GO) analysis of GPPGs and BPPGs.

Table S5. Gene set enrichment analysis (GSEA) results.

Table S6. List of cancer types from the TCGA database in the pan-cancer analysis.
